# Bioinformatics analysis revealing prognostic significance of *RRM2* gene in breast cancer

**DOI:** 10.1042/BSR20182062

**Published:** 2019-04-09

**Authors:** Wei-xian Chen, Liang-gen Yang, Ling-yun Xu, Lin Cheng, Qi Qian, Li Sun, Yu-lan Zhu

**Affiliations:** 1 Department of Breast Surgery, The Affiliated Changzhou No. 2 People’s Hospital of Nanjing Medical University, Changzhou 213000, Jiangsu Province, China; 2 Graduate School of Nanjing Medical University, Nanjing 210000, Jiangsu Province, China

**Keywords:** Biomarker, Breast cancer, Prognosis, RRM2

## Abstract

**Background:** Ribonucleotide reductase M2 subunit (*RRM2*) plays vital roles in many cellular processes such as cell proliferation, invasiveness, migration, angiogenesis, senescence, and tumorigenesis. However, the prognostic significance of *RRM2* gene in breast cancer remains to be investigated. **Methods:** *RRM2* expression was initially evaluated using the Oncomine database. The relevance between *RRM2* level and clinical parameters as well as survival data in breast cancer was analyzed using the Kaplan–Meier Plotter, PrognoScan, and Breast Cancer Gene-Expression Miner (bc-GenExMiner) databases. **Results:** *RRM2* was overexpressed in different subtypes of breast cancer patients. Estrogen receptor (ER) and progesterone receptor (PR) were negatively correlated with *RRM2* expression. Conversely, the Scarff–Bloom–Richardson (SBR) grade, Nottingham prognostic index (NPI), human epidermal growth factor receptor-2 (HER-2) status, nodal status, basal-like status, and triple-negative status were positively related to *RRM2* level in breast cancer samples with respect to normal tissues. Patients with increased *RRM2* showed worse overall survival, relapse-free survival, distant metastasis-free survival, disease-specific survival, and disease-free survival. *RRM2* also exerted positive effect on metastatic relapse event. Besides, a positive correlation between *RRM2* and *KIF11* genes was confirmed. **Conclusion:** Bioinformatics analysis revealed that *RRM2* might be used as a predictive biomarker for prognosis of breast cancer. Further studies are needed to more precisely elucidate the value of *RRM2* in evaluating breast cancer prognosis.

## Introduction

Breast cancer is the most frequently diagnosed tumor and a leading cause of cancer-related deaths among women worldwide [1]. Early diagnosis and treatment strategies including surgery, chemotherapy, radiotherapy, endocrine agents, and biological targeting agents have reduced patient morbidity and mortality; however, the prognosis of breast cancer remains poor. While clinical, pathological, and molecular features are widely used for establishing prognostics and predicting outcomes, finding more sensitive and specific biomarkers as surrogates of these features is the current trend in breast cancer research [2].

Ribonucleotide reductase M2 subunit (*RRM2*), a rate-limiting enzyme for DNA synthesis and repair, displays vital roles in many critical cellular processes such as cell proliferation, invasiveness, migration, angiogenesis, and senescence [3]. *RRM2* is frequently overexpressed in various malignancies and functions like a tumor driver [4–8]. Accumulating evidence has suggested that targeting *RRM2* may be a novel strategy for cancer treatment. For example, *RRM2* protected glioblastoma cells from endogenous replication stress, DNA damage, and apoptosis; *RRM2* inhibition sensitized glioblastoma cells to agent treatment [9]. Knockdown of *RRM2* attenuated melanoma growth both *in vitro* and *in vivo*, which correlated with maintenance of senescence-associated cell-cycle arrest [10]. In terms of breast cancer, both genetic suppression by RNA interference approach and pharmacological inhibition by small molecular antagonist of *RRM2* gene significantly reversed tamoxifen-resistant cell proliferation, reduced cell motility, activated pro-apoptotic pathways, and decreased tumor growth [11–13]. Moreover, it was reported that *RRM2* was associated with chemoresistance of breast cancer cells to adriamycin; suppression of *RRM2* synthesis could enhance the chemosensitivity to toxic insult [14]. Taken together, these findings suggest that *RRM2* may act not only as an oncogene, but also as a promising prognostic biomarker and potential therapeutic target in cancer.

Therefore, in the present study, we evaluated the significance of *RRM2* gene expression in breast cancer by using bioinformatics analysis of the clinical parameters and survival data in several large online databases.

## Materials and methods

### Oncomine

The Oncomine (http://www.oncomine.org), an online database containing microarray expression data from a variety of human cancers, was used to determine the level of *RRM2* in breast cancer patients and normal individuals with the threshold of fold change ≥ 2, *P*-value ≤ 1E-4, and gene rank ≥ top 10% [15]. Gene co-expressed with *RRM2* was analyzed and displayed as a heat map.

### Breast Cancer Gene-Expression Miner

The Breast Cancer Gene-Expression Miner v4.1 (bcGenExMiner v4.1, http://bcgenex.centregauducheau.fr/BC-GEM), a mining tool of published annotated genomics data, was utilized to evaluate the association between *RRM2* gene and clinical parameters, as well as the relevance with metastatic relapse event [16,17]. The correlation between *RRM2* and *KIF11* were generated using the correlation module.

### PrognoScan

The PrognoScan (http://www.prognoscan.org/) is a large database with clinical annotation and a web-based tool for assessing the biological relationship between gene expression and prognostic information including overall survival, relapse-free survival, distant metastasis-free survival, disease-specific survival, and disease-free survival in breast cancer patients [18]. Cox *P*-values and hazard ratio (HR) with 95% confidence intervals were calculated automatically.

### Kaplan–Meier Plotter

The Kaplan–Meier Plotter (http://kmplot.com/analysis/), a platform containing gene expression information and survival data of 5143 clinical breast cancer patients, was applied to verify the prognostic value of *RRM2* gene in overall survival, relapse-free survival, and distant metastasis-free survival [19].

### UCSC Xena

The heat map of *RRM2* and *KIF11* in the same patient cohort were constructed by data mining in TCGA Breast Cancer using the UCSC Xena browser (http://xena.ucsc.edu/).

## Results

### Increased expression of *RRM2* gene in breast cancer patients

**Figure 1:**
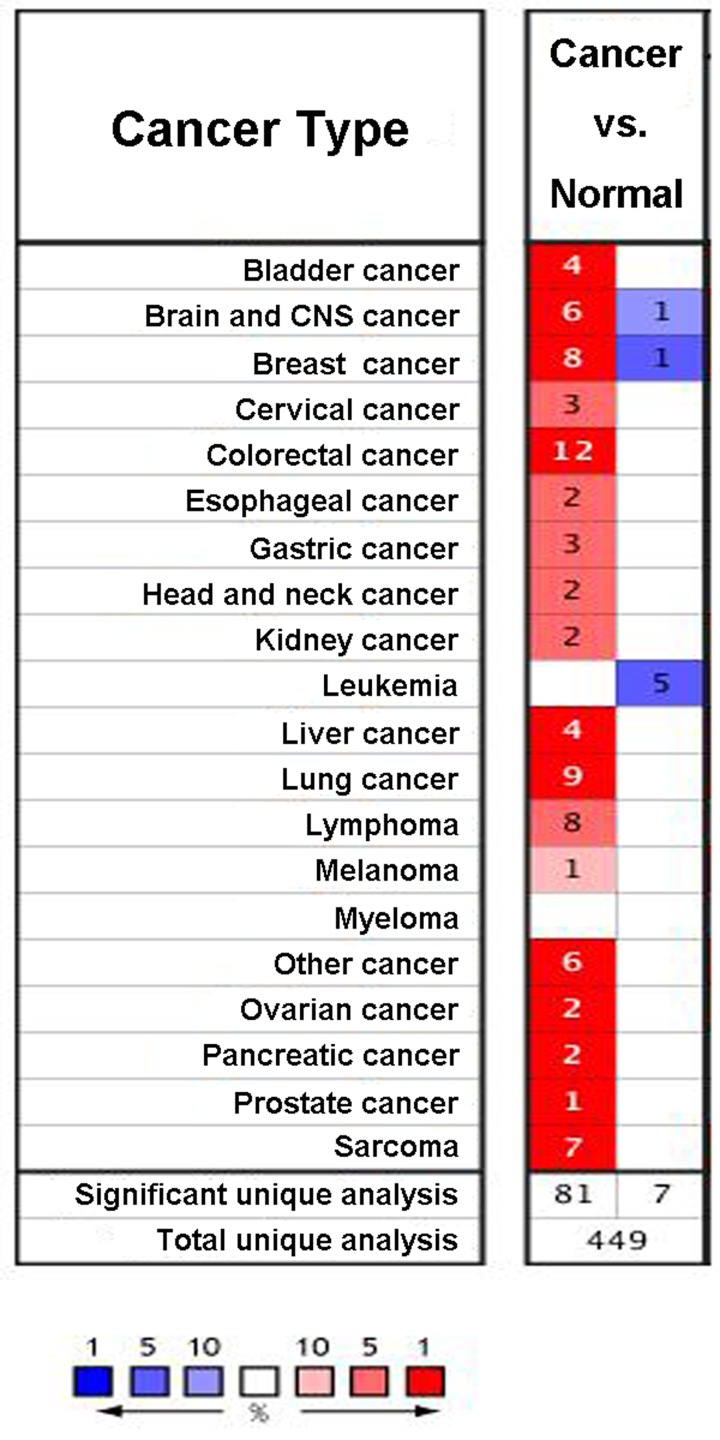
Expression of *RRM2* gene in 20 types of malignant tumor and corresponding normal tissues using the Oncomine database Red and blue represent the numbers of datasets with statistically significant (*P*<0.05) increased and decreased levels of *RRM2* gene, respectively. Cell color is determined by the best gene rank percentile for the analyses within the cell, and the gene rank was analyzed by percentile of target genes in the top of all genes measured by each study.

**Figure 2:**
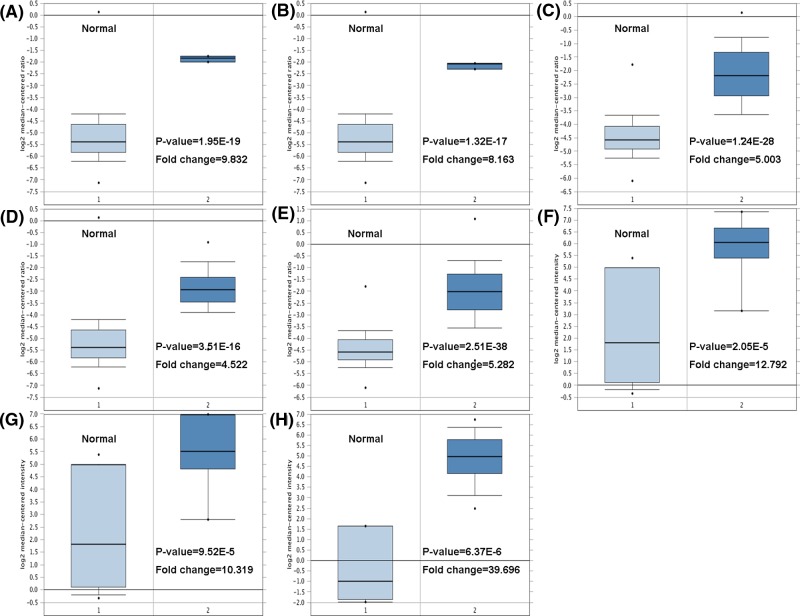
Box plot comparing *RRM2* expression in normal individuals and breast cancer patients derived from the Oncomine database Analysis is shown for male breast carcinoma (**A**), intraductal cribriform breast adenocarcinoma (**B**), invasive breast carcinoma (**C**), invasive lobular breast carcinoma (**D**), invasive ductal breast carcinoma (**E**), ductal breast carcinoma *in situ* (**F**), invasive ductal breast carcinoma epithelia (**G**), and ductal breast carcinoma (**H**). * stands for the maximum and minimum values.

We first checked the expression of *RRM2* gene in 20 types of malignant tumor using the Oncomine database. Increased level of *RRM2* (red) was observed in gastrointestinal cancers, gynecological cancers, urogenital cancers, and breast cancer (Figure 1). Our analysis also revealed that *RRM2* was significantly higher expressed in male breast carcinoma, intraductal cribriform breast adenocarcinoma, invasive breast carcinoma, invasive lobular breast carcinoma, invasive ductal breast carcinoma, ductal breast carcinoma *in situ*, invasive ductal breast carcinoma epithelia, and ductal breast carcinoma, compared with the corresponding normal tissues (Figure 2A–H and Table 1).

**Table 1:** *RRM2* expression in different subtypes of breast cancer and normal tissues using the Oncomine database

Breast cancer subtype	*P*-value*	*t*test	Fold change	Patient number	Reference
Male breast carcinoma	1.95E-19	19.864	9.832	3	TCGA
Intraductal cribriform breast adenocarcinoma	1.32E-17	18.111	8.163	3	TCGA
Invasive breast carcinoma	1.24E-28	14.159	5.003	76	TCGA
Invasive lobular breast carcinoma	3.51E-16	9.962	4.522	36	TCGA
Invasive ductal breast carcinoma	2.51E-38	20.624	5.282	389	TCGA
Ductal breast carcinoma *in situ* epithelia	2.05E-5	5.180	12.792	9	PMID: 19187537
Invasive ductal breast carcinoma epithelia	9.52E-5	4.513	10.319	9	PMID: 19187537
Ductal breast carcinoma	6.37E-6	9.800	39.696	40	PMID: 16473279

*Statistical significance was determined by the Student’s *t*test.

### *RRM2* expression and clinical parameters in breast cancer patients

**Figure 3:**
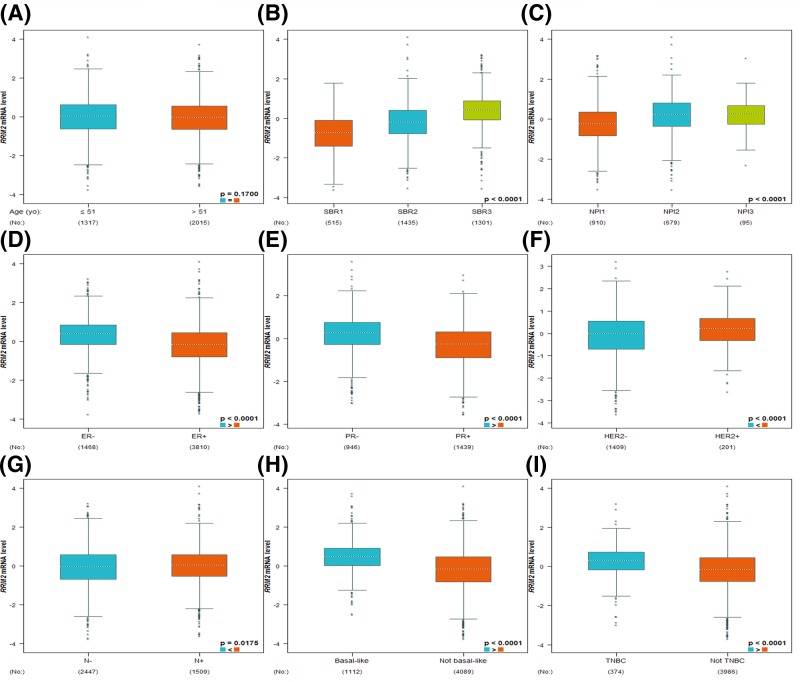
Box plot evaluating *RRM2* expression among groups of patients according to different clinical parameters using the bc-GenExMiner software Analysis is shown for age (**A**), SBR (**B**), NPI (**C**), ER (**D**), PR (**E**), HER-2(**F**), nodal status (**G**), basal-like status (**H**), and triple-negative status (**I**).

By using the bc-GenExMiner online tool, we next compared *RRM2* expression among groups of patients, according to different clinical parameters. Regarding age, there was no significant difference between ≤51- and >51-year group (Figure 3A and Table 2). The Scarff–Bloom–Richardson (SBR) is a histological grade that evaluates tubule formation, nuclear characteristics of pleiomorphism, and mitotic index. The Nottingham Prognostic Index (NPI) has been validated to stratify patients into additional prognostic groups according to tumor size, lymph node stage, and tumor grade. Breast cancer patients with more advanced SBR grade and NPI tended to express higher *RRM2* gene (Figure 3B,C). Estrogen receptor (ER) and progesterone receptor (PR) status were negatively associated with *RRM2* expression (Figure 3D,E and Table 2). Conversely, human epidermal growth factor receptor-2 (HER-2) status was confirmed to correlate positively with *RRM2* expression (Figure 3F and Table 2). Breast cancer patients with positive nodal status (N) showed increased level of *RRM2* than those with negative nodal status (Figure 3G and Table 2). Besides, we found that *RRM2* was strongly elevated in basal-like subtype with respect to non-basal-like subtype; the same pattern of change was also observed in triple-negative breast cancer (TNBC) patients (Figure 3H,I and Table 2).

**Table 2:** Relationship between *RRM2* expression and clinical parameters of breast cancer patients using the bc-GenExMiner database

Variables	Patient number	*RRM2* mRNA	*P*-value*
Age (years)			0.1700
≤51	1317	−	
>51	2015	−	
ER			<0.0001
Negative	1468	Increased	
Positive	3810	−	
PR			<0.0001
Negative	946	Increased	
Positive	1439	−	
HER-2			<0.0001
Negative	1409	−	
Positive	201	Increased	
Nodal status			0.0175
Negative	2447	−	
Positive	1509	Increased	
Basal-like status			<0.0001
Non-basal-like	4089	−	
Basal-like	1112	Increased	
Triple-negative status			<0.0001
Non-triple-negative	3986	−	
Triple-negative	374	Increased	

*Statistical significance was determined by the Welch’s test.

### *RRM2* expression and prognosis in breast cancer patients

**Figure 4:**
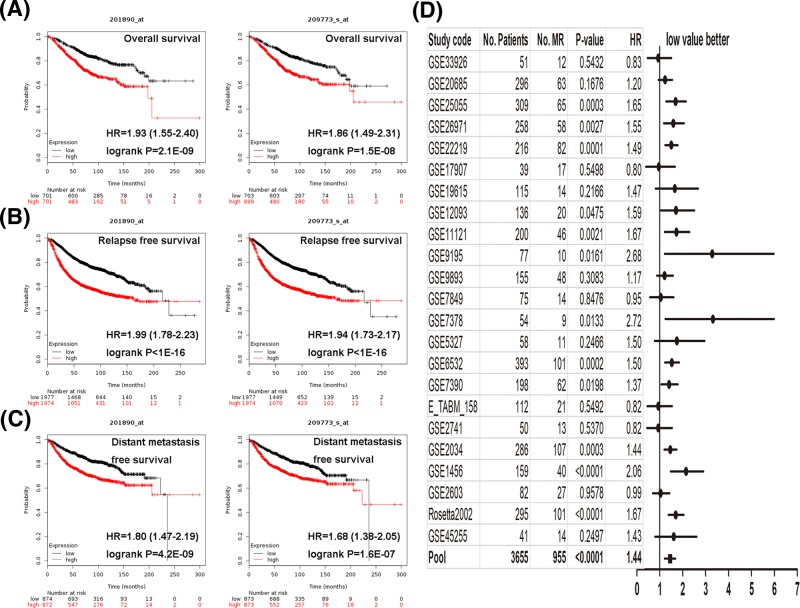
Survival curve and forest plot evaluating the prognostic value of *RRM2* Analysis is shown for overall survival (**A**), relapse-free survival (**B**), distant metastasis-free survival (**C**) using the Kaplan–Meier Plotter, and forest plot of metastatic relapse event using the bc-GenExMiner database (**D**).

We then investigated the prognostic value of *RRM2* gene. The Kaplan–Meier curves indicated that lower level of *RRM2* correlated with preferable overall survival (Figure 4A). While breast cancer patients with up-regulated *RRM2* demonstrated worse relapse-free survival (Figure 4B), patients with decreased *RRM2* expression presented better distant metastasis-free survival (Figure 4C). Furthermore, *RRM2* exerted positive effect on metastatic relapse event, as suggested by the forest plot using the bc-GenExMiner tool (Figure 4D). The PrognoScan database showed that overexpression of *RRM2* was significantly associated with inferior overall survival, relapse-free survival, distant metastasis-free survival, disease-specific survival, and disease-free survival (Table 3).

**Table 3:** *RRM2* expression and survival data of breast cancer patients using the PrognoScan database

Dataset	Probe name	End point	Patient number	Cox *P*-value	HR
GSE12276	209773_s_at	Relapse-free survival	204	0.001805	1.36 [1.12–1.65]
GSE6532-GPL570	209773_s_at	Relapse-free survival	87	0.025415	1.39 [1.04–1.87]
GSE6532-GPL570	209773_s_at	Distant metastasis-free survival	87	0.025415	1.39 [1.04–1.87]
GSE9195	209773_s_at	Relapse free survival	77	0.029912	2.01 [1.07–3.78]
GSE9195	209773_s_at	Distant metastasis-free survival	77	0.027181	2.30 [1.10–4.82]
GSE11121	209773_s_at	Distant metastasis-free survival	200	0.001108	1.99 [1.32–3.02]
GSE2034	209773_s_at	Distant metastasis-free survival	286	0.001001	1.64 [1.22–2.20]
GSE1456-GPL96	209773_s_at	Overall survival	159	0.000074	2.41 [1.56–3.73]
GSE1456-GPL96	209773_s_at	Relapse-free survival	159	0.000028	2.53 [1.64–3.90]
GSE1456-GPL96	209773_s_at	Disease-specific survival	159	0.000014	3.23 [1.90–5.47]
GSE7378	201890_at	Disease-free survival	54	0.021327	1.99 [1.11–3.59]
GSE7378	209773_s_at	Disease-free survival	54	0.013458	2.36 [1.19–4.67]
E-TABM-158	209773_s_at	Disease-specific survival	117	0.026992	0.71 [0.53–0.96]
GSE3494-GPL96	209773_s_at	Disease-specific survival	236	0.000122	2.07 [1.43–3.00]
GSE4922-GPL96	209773_s_at	Disease-free survival	249	0.000007	1.96 [1.46–2.63]
GSE2990	209773_s_at	Relapse-free survival	62	0.016824	1.73 [1.10–2.70]
GSE2990	209773_s_at	Distant metastasis-free survival	54	0.012179	2.04 [1.17–3.56]
GSE7390	209773_s_at	Overall survival	198	0.012109	1.35 [1.07–1.70]
GSE7390	209773_s_at	Distant metastasis-free survival	198	0.049656	1.24 [1.00–1.54]

### Co-expression of *RRM2* gene

**Figure 5:**
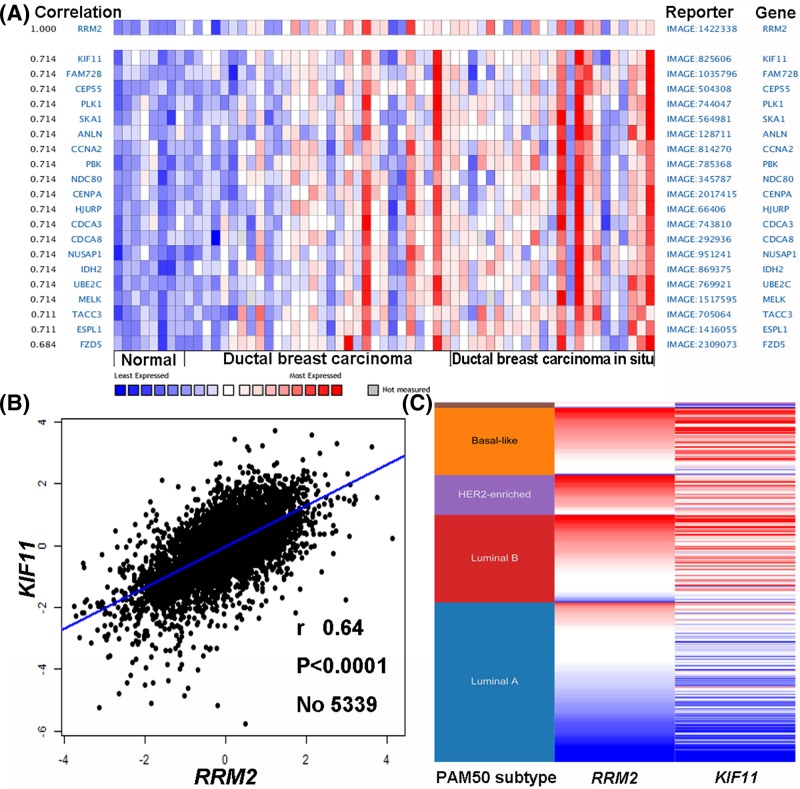
Co-expression of *RRM2* gene (**A**) Co-expression profile of *RRM2* identified using the Oncomine database. (**B**) Correlation between *RRM2* and *KIF11* expression in breast cancer analyzed using the bc-GenExMiner software. (**C**) Heat map of *RRM2* and *KIF11* expression across PAM50 breast cancer subtypes in the TCGA database obtained from the UCSC Xena web-based tool.

To further investigate the underlying regulation of *RRM2* in breast cancer, data mining of the co-expression of *RRM2* gene was performed using the Oncomine database. The co-expression profile of *RRM2* was identified with a large cluster of 1802 genes across 61 breast carcinomas, and *KIF11* is a principal correlated gene (Figure 5A). Data mining in bc-GenExMiner revealed a positive correlation between *RRM2* and *KIF11* (Figure 5B). By comparing the *RRM2* and *KIF11* expression heat map derived from the UCSC Xena web-based tool, it was confirmed that *RRM2* expression gradually elevated with increasing *KIF11* transcript level, which was determined among a 50-gene qPCR assay (PAM50) breast cancer subtypes in TCGA database (Figure 5C). These data indicated that *RRM2* could be associated with the *KIF11* signaling pathways in breast cancer.

## Discussion

*RRM2* plays vital roles in diverse cellular functions such as cell proliferation, invasiveness, migration, angiogenesis, senescence, and tumorigenesis [3]. It was reported that *RRM2* was associated with resistance of breast cancer cells to chemotherapy and endocrine agents and that targeting *RRM2* may be a novel strategy for cancer treatment [11–14]. However, the significance of *RRM2* expression in prognosis of breast cancer remains largely unclear.

In the present work, we analyzed the expression profile of *RRM2* by Oncomine database. *RRM2* gene was higher expressed in male breast carcinoma, intraductal cribriform breast adenocarcinoma, invasive breast carcinoma, invasive lobular breast carcinoma, invasive ductal breast carcinoma, ductal breast carcinoma *in situ*, invasive ductal breast carcinoma epithelia, and ductal breast carcinoma patients, with respect to normal individuals. By using the bc-GenExMiner online tool, we found that ER and PR were negatively correlated with *RRM2* expression. Conversely, SBR, NPI, HER-2 status, nodal status, basal-like status, and triple-negative status were positively related to *RRM2* level in breast cancer samples with respect to normal tissues. As known to all, patients with ER or PR negative, nodal positive, HER-2 positive, basal-like or triple-negative status generally display an unsatisfied therapeutic response and worse clinical outcome. Therefore, our results suggested that lower expression of *RRM2* may predict a better prognosis of breast cancer.

We further investigated the prognostic value of *RRM2* in breast cancer using the Kaplan–Meier Plotter, PrognoScan, and bc-GenExMiner databases. Patients with increased *RRM2* showed worse overall survival, relapse-free survival, distant metastasis-free survival, disease-specific survival, and disease-free survival. Additionally, high *RRM2* expression was correlated with an increased risk of metastatic relapse event, as suggested by the forest plot. These findings collectively demonstrated that the level of *RRM2* might be a useful predictive biomarker for prognosis of breast cancer. We finally analyzed the co-expression of *RRM2* using the Oncomine, bc-GenExMiner, and UCSC Xena web-based tools and confirmed that *KIF11* gene was positively correlated with *RRM2* expression. *KIF11*, a molecular motor protein involved in mitosis, was critical for proliferation and self-renewal in chemoresistant breast cancer cells [20]. *KIF11* knockdown inhibited tumor growth both *in vitro* and *in vivo*, and its expression was responsible for shorter survival time [21]. Thus, our data indicated that *RRM2* might contribute to breast cancer progression and drug insensitivity associated with *KIF11* expression.

In summary, the present bioinformatics analysis showed that *RRM2* was overexpressed in breast cancer patients with respect to normal tissues and was associated with a worse survival. *RRM2* could be used as a predictive biomarker for prognosis of breast cancer with co-expressed *KIF11* gene. Further studies are needed to more precisely elucidate the value of *RRM2* in evaluating breast cancer prognosis.

## Abbreviations

bc-GenExMiner: Breast Cancer Gene-Expression Miner
ER: estrogen receptor
HER-2: human epidermal growth factor receptor-2
NPI: Nottingham Prognostic Index
PR: progesterone receptor
RRM2: ribonucleotide reductase M2 subunit
SBR: Scarff–Bloom–Richardson
